# Recurrence of Postoperative Atrial Fibrillation After Cardiac Surgery: Insights from a Tertiary Follow-Up Clinic

**DOI:** 10.1016/j.cjco.2025.09.016

**Published:** 2025-10-10

**Authors:** Rubani S. Suri, Emilie P. Belley-Côté, Siobhan M. Baigent, Nicole P. Veloce, Muneeb Ahmed, P.J. Devereaux, Jeff S. Healey, Richard P. Whitlock, William F. McIntyre

**Affiliations:** a Department of Medicine, McMaster University, Hamilton, Ontario, Canada; b Population Health Research Institute, McMaster University, Hamilton, Ontario, Canada; c Hamilton Health Sciences, McMaster University, Hamilton, Ontario, Canada; d Department of Medicine, University of Toronto, Toronto, Ontario, Canada; e Department of Surgery, McMaster University, Hamilton, Ontario, Canada

**Keywords:** Atrial fibrillation, cardiac surgery, electrocardiography, monitoring, post-operative

## Abstract

**Background:**

New-onset postoperative atrial fibrillation (POAF) complicates 30% of cardiac surgeries. Although POAF is often transient, structured follow-up care of patients with POAF may identify those with paroxysmal or persistent atrial fibrillation (AF) who will benefit from evidence-based therapies.

**Methods:**

This retrospective study includes patients seen in a clinic dedicated to patients with POAF after cardiac surgery between 2020 and 2024. Per the clinic’s operating procedure, patients wore a 14-day continuous ambulatory electrocardiogram (ECG) monitor fpr 2 months after surgery and were assessed thereafter in clinic. The primary outcome was recurrent AF lasting ≥ 30 seconds, captured by 14-day continuous ambulatory ECG or during clinical care.

**Results:**

The cohort included 881 patients, with a mean age of 68 ± 9 years, and a median **C**ongestive Heart Failure, **H**ypertension, **A**ge ≥ 75 Years, **D**iabetes Mellitus, **S**troke, **V**ascular Disease, **A**ge 65 to 74 Years, **S**ex **C**ategory (CHA_2_DS_2_-VASc) score of 2 (interquartile range [IQR] 1-3); 529 patients (60.0%) underwent isolated coronary artery bypass grafting. At discharge, 798 patients (90.6%) were prescribed amiodarone, and 435 (49.4%) were prescribed oral anticoagulation. The mean time between discharge and 14-day continuous ambulatory ECG monitor was 72 days (IQR 61-84). AF recurrence was detected in 94 patients (10.7%); 30 patients (36.1%) were not receiving oral anticoagulation at the time of recurrence. Among patients with recurrence detected by 14-day continuous ambulatory ECG, the median duration was 10 hours (IQR 2-253). Left atrial volume index was the only independent predictor of AF recurrence. Following the clinic visit, oral anticoagulation was continued in 122 patients (28.2%).

**Conclusions:**

Among patients with POAF following cardiac surgery, 1 in 10 have AF recurrence, as determined by a structured 14-day continuous ambulatory ECG monitor utilized 2-3 months postoperatively.

## Central Illustration

**Figure:**
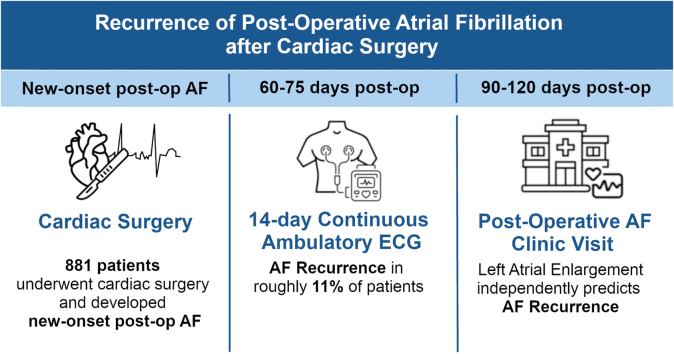

## Résumé

**Contexte:**

La fibrillation auriculaire postopératoire (FAPO) d'apparition nouvelle complique 30 % des chirurgies cardiaques. Bien que la FAPO soit souvent transitoire, un suivi structuré des patients présentant une FAPO peut identifier ceux qui souffrent de fibrillation auriculaire (FA) paroxystique ou persistante et qui pourraient bénéficier de traitements fondés sur des données probantes.

**Méthodologie:**

Cette étude rétrospective porte sur des patients suivis dans une clinique spécialisée dans la prise en charge des patients atteints de FAPO après une chirurgie cardiaque réalisée entre 2020 et 2024. Conformément au mode opératoire de la clinique, les patients portaient un moniteur d'électrocardiogramme (ECG) ambulatoire continu pendant 14 jours, 2 mois après l'opération, puis ont été évalués en clinique. Le critère d'évaluation principal était la récidive de la FA pour une durée ≥ 30 secondes, détectée par le moniteur d'ECG ambulatoire continu de 14 jours, ou pendant les soins cliniques.

**Résultats:**

La cohorte comprenait 881 patients, d'âge moyen de 68 ± 9 ans, avec un score CHA_2_DS_2_-VASc médian de 2 (intervalle interquartile [IIQ] 1-3); 529 patients (60,0 %) ont subi un pontage aortocoronarien isolé. À leur sortie de l'hôpital, 798 (90,6 %) patients se sont vu prescrire de l'amiodarone et 435 (49,4 %) une anticoagulation orale. Le délai moyen entre la sortie de l'hôpital et le monitoring ECG ambulatoire continu de 14 jours était de 72 jours (IIQ 61-84). Une récidive de FA a été détectée chez 94 patients (10,7 %); 30 patients (36,1 %) ne recevaient pas d'anticoagulants oraux au moment de la récidive. Parmi les patients chez lesquels une récidive a été détectée par ECG ambulatoire continu pendant 14 jours, la durée médiane était de 10 heures (IIQ 2-253). L'indice de volume auriculaire gauche était le seul facteur prédictif indépendant de récidive de FA. Après la consultation, l'anticoagulation orale a été poursuivie chez 122 patients (28,2 %).

**Conclusions:**

Parmi les patients présentant une FAPO après une chirurgie cardiaque, 1 sur 10 présente une récidive de FA lors d'un suivi structuré à l'aide d'un moniteur ECG ambulatoire continu pendant 14 jours, réalisé 2 à 3 mois après l'opération.

Atrial fibrillation (AF) is the most common complication of cardiac surgery, occurring in 20%-55% of patients who have cardiac surgery.^1^^,^^2^ Postoperative AF (POAF) refers to AF detected within the first 4 weeks after cardiac surgery in patients without a history of AF.^1^^,^^3^ POAF is associated with a 2-fold increased risk of all-cause 30-day mortality, and patients with POAF spend, on average, 4 extra days in the hospital after surgery.^1^, ^2^, ^3^, ^4^, ^5^

Although POAF is usually transient, it may confer a long-term risk for clinical AF, stroke, heart failure, and mortality.^6^ AF occurring in the postsurgical setting presents uncertainty regarding whether the rhythm was secondary to the transient physiological triggers associated with cardiac surgery or is the first presentation of paroxysmal or persistent AF.^6^^,^^7^ This distinction between self-limiting arrhythmia and an ongoing pattern of AF is critical for clinicians, who must decide if POAF can be dismissed as a reversible phenomenon, or signals a need for long-term anticoagulation. Our current understanding of the natural history of POAF is limited.^8^ Several studies have investigated the recurrence of POAF after cardiac surgery using electrocardiogram (ECG) monitoring, but these studies are limited by their small sample size or lack of structured follow-up, restricting their generalizability.^9^, ^10^, ^11^ The 2020 Canadian Cardiovascular Society guidelines recommend that patients with POAF be “followed indefinitely for the possible emergence of recurrent clinical AF,” but no specific data are available to guide monitoring intensity and frequency.^12^

The primary objective of this study was to estimate the rate of AF recurrence in cardiac surgery patients with new-onset POAF in a large cohort using 14-day continuous ambulatory ECG monitoring. The secondary objective was to explore clinical factors that are associated with AF recurrence.

## Material and Methods

### Study population

This retrospective observational study includes consecutive patients seen in the Cardiac Surgery Post-Operative Atrial Fibrillation Follow-up Clinic at the Hamilton General Hospital in Hamilton, Ontario, Canada from September 2020 to April 2024. The in-patient perioperative care team refers all patients who develop new-onset AF within the first 4 weeks after cardiac surgery to this clinic. Most patients are referred to this clinic at the time of hospital discharge, but the clinic also receives referrals from other healthcare providers who capture AF in the early postoperative period. Approximately 1500 cardiac surgeries are performed annually at the Hamilton General Hospital. Patients wear a 14-day continuous ambulatory ECG (Holter) monitor for approximately 2-3 months following surgery and are assessed in the clinic thereafter. This time interval was chosen based on recommendations for anticoagulation to continue for 60-90 days postoperatively.^13^ This clinic flowchart is depicted in the Central Illustration.

**Central Illustration:**
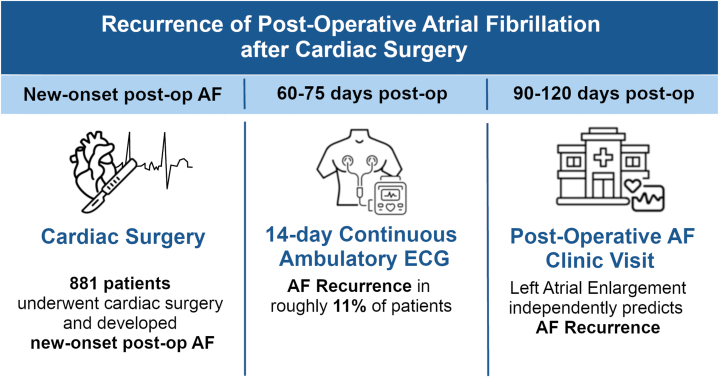
Recurrence of postoperative (post-op) atrial fibrillation (AF) after cardiac surgery. This figure summarizes the workflow of the POAF clinic. Following new-onset POAF at the time of cardiac surgery, patients wore a 14-day continuous ambulatory electrocardiogram (ECG) monitor 60-75 days postoperatively. AF recurrence was documented in 11% of patients. At 90-120 days postoperatively, follow-up clinic visits revealed that left atrial enlargement was an independent predictor of AF recurrence.

Eligible patients for this analysis had new-onset AF detected within the first 4 weeks following cardiac surgery, wore a 14-day continuous ambulatory ECG monitor, and attended the follow-up clinic. We used a 4-week cutoff to define postoperative AF based on prior studies that show that the incidence and prevalence of AF are both elevated during this period.^14^^,^^15^ AF recurrence was defined as AF lasting ≥ 30 seconds, detected either by the 14-day continuous ambulatory ECG or during clinical care. Eligible patients were captured through a review of clinic records.

The Hamilton Integrated Research Ethics Board approved this study.

### Data collection

We reviewed electronic medical charts retrospectively. We extracted data on patient information, medical history, medication at clinic visit and hospital discharge, surgery information, in-hospital AF, 14-day continuous ambulatory ECG, transthoracic echocardiogram, and clinic outcomes.

The primary objective of this study was to estimate AF recurrence captured by 14-day continuous ambulatory ECG monitoring. The secondary objective was to explore risk factors for developing recurrent AF after discharge.

### Statistical methods

We performed statistical analysis using R (R Foundation, Vienna, Austria). We express measurements as a mean ± standard deviation (X ± SD), and we used an independent samples *t*test to compare patients with vs without AF recurrence. For data that did not fit the normal distribution, we reported median with interquartile range (median, IQR) and used nonparametric tests. We express count data as frequencies and percentages (n, %), and we used χ^2^ testing to compare groups.

We used univariable and multivariable logistic regression analysis to analyze potential risk factors for AF recurrence. We considered a *P* value < 0.05 to be statistically significant. We prespecified the following variables for analysis: female biological sex, age, CHDS_2_-VA score (**C**ongestive Heart Failure, **H**ypertension, **A**ge ≥ 75 Years, **D**iabetes Mellitus, **S**troke, **V**ascular Disease, **A**ge 65 to 74 Years, **S**ex **C**ategory [CHA_2_DS_2_-VASc] score excluding sex and age), surgery other than coronary artery bypass grafting (CABG), and left atrial volume index (LAVI). These variables were selected *a priori*, based on previous work evaluating predictors of AF recurrence in noncardiac surgery.^16^ Each variable was assessed in a univariable analysis, and then was forced into a multivariable logistic regression model. We first modelled left atrial enlargement as a continuous variable to maximize statistical power. We prespecified that if this was statistically significant as a continuous variable in a multivariable model, we would run a univariable dichotomizing normal and mild left atrial enlargement vs moderate and severe enlargement. We categorized left atrial size using LAVI according to the 2015 American Society of Echocardiography chamber quantification guidelines, with normal to mild left atrial enlargement defined by an LAVI of 16-41 mL/m^2^ and moderate to severe enlargement defined by an LAVI of ≥ 42 mL/m^2^.^17^

We chose a binary outcome variable for recurrence, and coded “yes” as 1 and “no” as 0. We fitted a multivariable logistic regression model using the “glm()” function with a binomial link, including the following covariates: female biological sex, age, CHDS_2_-VA, surgical procedure other than isolated coronary artery bypass graft, and LAVI. We calculated odds ratios (ORs) and corresponding 95% confidence intervals (CIs). We conducted all analyses using base R functions, and no additional packages were required.

## Results

**Figure 1:**
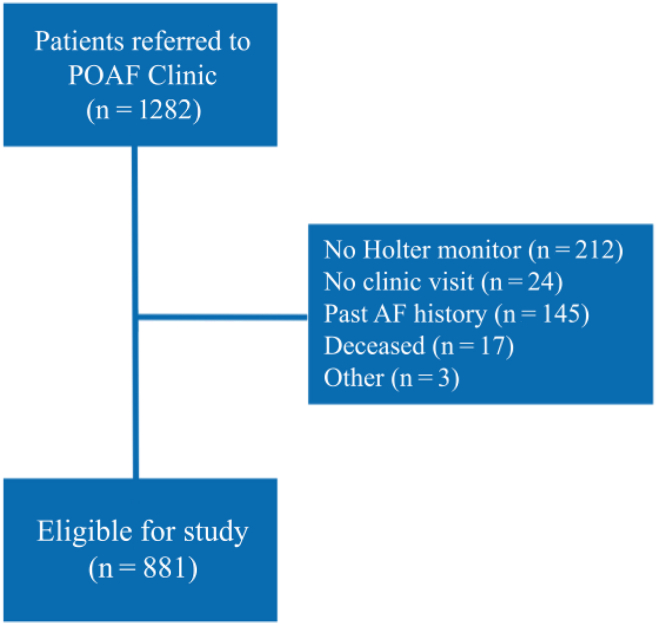
Postoperative atrial fibrillation (POAF) clinic visit summary.

A total of 881 patients underwent cardiac surgery, had POAF, wore a 14-day continuous ambulatory ECG monitor, and attended the POAF clinic (Fig. 1). Table 1 reports patient characteristics at index hospital admission. Of 881 patients undergoing cardiac surgery who were included in this study, 529 patients (60.0%) underwent isolated CABG, 120 patients (13.6%) underwent isolated valve surgery, 26 patients (3.0%) underwent isolated aortic surgery, 100 patients (11.3%) underwent combined CABG and valve surgery, 19 patients (2.2%) underwent combined CABG and aortic surgery, 57 patients (6.5%) underwent combined valve surgery and aortic surgery, and 30 patients (3.4%) underwent combined CABG, valve surgery, and aortic surgery. Eight patients (0.9%) underwent posterior pericardiotomy during their surgery. During the index hospitalization, treating clinicians attempted electrical cardioversion on 55 patients (6.2%); 798 patients (90.6%) were discharged on amiodarone. Nearly all patients were discharged in sinus rhythm—54 patients (6.1%) had AF documented as their final predischarge rhythm. Table 2 reports the baseline characteristics of patients as captured at time of clinic visit. The median CHA_2_DS_2_-VASc score was 2 (IQR = 1-3), and the mean LAVI was 36.4 mL/m^2^ ± 12.9.

**Table 1:** Characteristics of index admission

	n (%)∗
**Surgery characteristics**	
CABG	662 (75.1)
Isolated CABG	529 (80.4)
Number of grafts, mean ± SD	3.4 ± 1.1
Use of left internal mammary artery	553 (62.8)
Use of right internal mammary artery	2 (0.2)
Valvular surgery	307 (34.8)
Operation on aortic valve	234 (26.5)
Type of aortic valve surgery	
Repair	6 (2.6)
Replacement, bioprosthetic	215 (91.9)
Replacement, mechanical	10 (4.3)
Ross procedure	3 (1.3)
Operation on mitral valve	68 (7.7)
Operation on tricuspid valve	5 (0.6)
Aortic surgery	132 (14.9)
Type of aorta surgery	
Root repair or replacement	8 (6.1)
Ascending aortic root repair or replacement	112 (84.8)
Arch replacement	2 (1.5)
Descending thoracic aortic repair or replacement	1 (0.8)
Posterior pericardiotomy	8 (0.9)
**Other admission characteristics**	
Heart failure at admission	16 (1.8)
Acute coronary syndrome at admission	502 (57.0)
Discharge destination	
Home	805 (91.4)
Long-term care	6 (0.7)
Rehabilitation facility	70 (7.9)
Duration of index POAF episode, h	370 (42.0)
0–24	160 (18.2)
> 24	106 (12.0)
More than one episode of POAF during index hospitalization	136 (15.4)
Perioperative cardioembolic stroke	5 (0.6)
Cardioversion in-hospital	852 (96.7)
Electrical cardioversion	55 (6.2)
TEE done prior to electrical cardioversion	47 (5.3)
Pharmacologic cardioversion	753 (85.5)
Amiodarone	743 (98.7)
Agent other than amiodarone	10 (1.1)
Discharged in sinus rhythm	827 (93.9)

CABG, coronary artery bypass graft; POAF, postoperative atrial fibrillation; SD, standard deviation; TEE, transesophageal echocardiography.

∗ Values are n (%) unless otherwise indicated.

**Table 2:** Baseline characteristics of patients∗

Characteristic	Value
Age, y	68.3 ± 8.7
Sex	
Female	194 (22.0)
Body mass index, kg/m^2^	28.8 ± 5.6
Previous cardiac surgery	7 (0.8)
CHA_2_DS_2_-VASc score†	2 (1–3)
Congestive heart failure	40 (4.5)
Hypertension	585 (66.4)
Diabetes mellitus	269 (30.5)
Stroke	27 (3.1)
Systemic arterial embolism	1 (0.1)
Transient ischemic attack	24 (2.7)
Peripheral arterial disease	53 (6.0)
Coronary artery disease	234 (26.6)
Known aortic plaque	35 (3.9)
Myocardial infarction	62 (7.0)
Sleep apnea	124 (14.1)
Rheumatic heart disease	15 (1.7)
Deep vein thrombosis	11 (1.2)
Obstructive or restrictive lung disease	58 (6.6)
Mean left ventricular ejection fraction, %	54.3 ± 11.9
Mean left atrium diameter, cm	4.2 ± 2.9
Mean left atrium volume index, mL/m^2^	36.4 ± 12.9
Left atrial size	
Normal	349 (39.6)
Mildly enlarged	114 (12.9)
Moderately enlarged	83 (9.4)
Severely enlarged	77 (8.7)

Values are n (%), mean ± standard deviation, or median (interquartile range).

∗ Captured at time of clinic visit.

† The CHA_2_DS_2_-VASc is calculated based on presence of hypertension (1 point), heart failure (1 point), age ≥ 75 years (2 points), age 65-74 years (1 point), diabetes mellitus (1 point), previous stroke and/or transient ischemic attack (2 points), female sex (1 point), and vascular disease (1 point).^29^

Patients began wearing a 14-day continuous ambulatory ECG monitor after a median of 72 days (IQR = 61-84) after hospital discharge. An average of 85.6% (± 14.6%) of the ECG monitoring time was analyzable. In total, 334 (37.4%) reported having at least one symptomatic event while wearing the monitor. A total of 94 patients (10.7%) had AF recurrence. Of 94 patients with AF recurrence, 16 (19.3%) had reported symptoms. This number includes 83 (88.3%) with AF recurrence reported on their 14-day continuous ambulatory ECG; 11 patients (11.7%) had AF detected during clinical care. Among the 79 patients who were not discharged on amiodarone, 9 patients (11.4%) had AF recurrence detected on the 14-day continuous monitoring. Of 55 patients who underwent an electrical cardioversion during their index hospitalization, 9 patients (16.4%) had AF recurrence. Among patients with AF recurrence, the median AF burden (defined as % of analyzable time in AF) was 7.0% (IQR 0.7-97.8), with 28 patients (33.7%) having an AF burden > 50%. Among patients with recurrent AF, the median AF duration was 10 hours (IQR 1.7-253.1).

Table 3 presents a comparison of medications at the time of hospital discharge and clinic visit. Among 94 patients with AF recurrence, 53 (63.8%) were on oral anticoagulation (OAC) at the time of AF recurrence on ECG monitoring. At the time of clinic visit, 433 patients (49.1%) were on OAC. Following the clinic visit, OAC was discontinued in 311 patients (71.8%) and was continued in 122 patients (28.2%). OAC was initiated in 14 patients (1.6%). Reasons for patients continuing or starting OAC included AF (60.6%), stroke occurring between surgery and the clinic visit (13.4%), deep vein thrombosis (5.1%), pulmonary embolism (2.7%), mechanical heart valve (12.4%), or left ventricular thrombus (5.8%). A total of 65 patients (7.4%) were on amiodarone at the time of clinic visit. Amiodarone was discontinued in 50 patients (76.9%). Amiodarone was started in 5 patients (0.6%); in all cases, this was to facilitate cardioversion in a patient with persistent AF.

**Table 3:** Medication at hospital discharge and clinic visit

Medication	At hospital discharge	At clinic visit
Antiarrhythmic agent		
Amiodarone	798 (90.6)	65 (7.4)
Beta-blocker	796 (90.4)	754 (85.6)
Digoxin	5 (0.6)	4 (0.5)
Dihydropyridine calcium channel blocker	164 (18.6)	157 (17.8)
Rate-controlling calcium channel blocker	1 (0.1)	3 (0.3)
Dronedarone	0 (0)	1 (0.1)
Sotalol	0 (0)	2 (0.2)
Antiplatelet agent		
Aspirin	712 (80.8)	679 (77.1)
Clopidogrel	290 (32.9)	288 (32.6)
Prasugrel	0 (0)	0 (0)
Ticagrelor	3 (0.3)	3 (0.3)
Oral anticoagulation	435 (49.4)	433 (49.1)
Warfarin	371 (42.1)	328 (37.2)
Dabigatran	29 (3.3)	30 (3.4)
Rivaroxaban	6 (0.7)	13 (1.5)
Apixaban	26 (3.0)	48 (5.4)
Edoxaban	3 (0.3)	14 (1.6)
Heparin	1 (0.1)	1 (0.1)
Angiotensin-converting enzyme inhibitor	145 (16.5)	192 (21.8)
Angiotensin receptor blocker	125 (14.2)	154 (17.5)
Statin	735 (83.4)	727 (82.5)
Sodium-glucose cotransporter-2 inhibitor	136 (15.4)	173 (19.6)
Insulin	92 (10.4)	81 (9.2)
Oral anti-hyperglycemic	175 (19.9)	178 (20.2)
Colchicine	76 (8.6)	29 (3.3)

Values are n (%).

A total of 106 patients (12.0%) were seen for a repeat clinic visit. Among these, 50 patients (53.2%) were on amiodarone at the time of first clinic visit and required a repeat continuous ambulatory ECG after it was discontinued. A total of 41 patients (43.6%) had AF present at the initial clinic visit and were scheduled for a 3-month follow-up evaluation. A total of 11 patients (11.7%) required a repeat visit for cardioversion, and 4 (4.3%) required a repeat visit to assess for AF ablation. A total of 4 patients (0.4%) had new AF recurrence diagnosed between the initial visit and the repeat visit, captured by a modality other than the 14-day continuous ambulatory ECG. At the repeat visit, OAC was started in 4 patients (3.7%), continued in 26 patients (24.5%), and stopped in 19 patients (17.9%).

Univariable and multivariable predictors of AF recurrence on the 14-day continuous ambulatory ECG are shown in Table 4. In an adjusted multivariable model, LAVI was the only independent predictor of AF recurrence. When tested in a univariable model, patients with a moderate or severe left atrial enlargement (ie, LAVI ≥ 42 mL/m^2^) were more likely to have AF recurrence, compared to patients with a normal left atrium or mild left atrial enlargement (OR: 2.4, 95% CI: 1.2-9.8). None of the following factors were found to be independently associated with AF recurrence: female biological sex; age; CHDS_2_-VA score (CHA_2_DS_2_-VASc excluding sex and age); and surgery type.

**Table 4:** Predictors of atrial fibrillation recurrence on a 14-day continuous ambulatory electrocardiogram monitor in patients with postoperative atrial fibrillation

Predictor	Univariable odds ratio (95% CI); *P*	Multivariable∗ odds ratio (95% CI); *P*
Female biological sex	1.3 (0.7–2.3); 0.3	1.3 (0.5–2.8); 0.6
Age, per y	1.0 (1.0–1.1); 0.1	1.0 (1.0–1.1); 0.1
CHDS_2_-VA,† per point	1.0 (0.9–1.2); 0.7	1.1 (0.9–1.3); 0.1
Surgical procedure other than isolated coronary artery bypass graft	1.2 (0.7–1.9); 0.5	1.2 (0.6–2.3); 0.6
Left atrial volume index, per 10 mL per squared body surface area	1.4 (1.1–1.6); 0.01	1.4 (1.1–1.6); 0.01

CI, confidence interval.

∗ Adjusted for all other variables in the table.

† **C**ongestive Heart Failure, **H**ypertension, **A**ge ≥ 75 Years, **D**iabetes Mellitus, **S**troke, **V**ascular Disease, **A**ge 65 to 74 Years, **S**ex **C**ategory (CHA_2_DS_2_-VASc) excluding age and sex.

## Discussion

Using records from a clinic focused on following patients with POAF after cardiac surgery, we estimated that approximately 11% of patients with POAF will have AF recurrence documented when they are followed using continuous ambulatory ECGs in a structured manner. We identified larger left atrial size as a predictor of AF recurrence on the 14-day continuous ambulatory ECG.

The incidence of AF recurrence following POAF has been reported previously, with studies generating a range of estimates using different monitoring strategies and follow-up periods. In a systematic review of 185 patients with new-onset POAF within 30 days of cardiac surgery who received an implantable loop recorder, we estimated the rate of AF recurrence at 3 months to be 17.8% (95% CI: 11.9%-23.2%).^18^ In a retrospective cohort study including 355 patients with POAF, William and colleagues documented that AF recurrence was detected in 29% after a mean of 4.7 + 2.4 years of routine clinical follow-up care.^19^ In a prospective Greek cohort study that used a 12-lead ECG and a 24-hour continuous ECG at 3, 6, and 12 months of follow-up care, investigators documented AF recurrence in 3.6% of POAF patients.^20^ We used 14-day continuous ambulatory ECG worn for around 70 days after surgery. We found an AF recurrence rate of 10.7%. Taken in context with the other studies, the AF detection rates likely could have been increased with longer follow-up or repeat monitoring, but this monitoring strategy might have identified a subset of higher-risk patients.

Among the variables we studied, left atrial size was the only independent predictor of AF recurrence on the 14-day continuous ambulatory ECG. Left atrial size has been identified as a strong predictor of AF detection in both follow-up of patients with POAF after noncardiac surgery and population-based studies.^16^^,^^21^^,^^22^ This finding supports the role of AF size as a marker for AF-sustaining substrate and reinforces the value of this parameter as a key risk marker for AF.^12^

In this study, patients who had recurrent AF detected were in AF for a median total of 10 hours (IQR 1.7-253.1). This length of time is greater than the 6-minute threshold associated with a risk for stroke seen in studies that used implantable cardiac monitors.^23^^,^^24^ A subanalysis in ASSERT (Asymptomatic Atrial Fibrillation and Stroke Evaluation in Pacemaker Patients and the Atrial Fibrillation Reduction Atrial Pacing Trial) suggested that this 6-minute cutoff also applies to 14-day continuous ambulatory ECG monitors.^25^ This AF duration is also comparable to the AF burden that has been documented in patients having AF ablation.^26^^,^^27^ This finding suggests that the AF detected in follow-up is prognostically important and that these patients are likely to benefit from OAC for stroke prevention, and possibly from rhythm control.

The 2020 Canadian Cardiovascular Society guidelines recommend that patients with POAF be “followed indefinitely for the possible emergence of recurrent clinical AF.” This recommendation implies that continued follow-up is needed after discharge from the POAF clinic. This study offers insights into a first step in the long-term management of patients who develop POAF following cardiac surgery, in line with these recommendations.^12^ A 14-day continuous ambulatory ECG monitor is available in many healthcare jurisdictions and is a readily available tool that can be used to risk stratify the large number of patients with POAF following cardiac surgery.

### Strengths and limitations

To our knowledge, this study is the largest to document the structured follow-up care of an unselected group of patients with POAF following cardiac surgery. We employed 14-day continuous ECG monitoring, which is readily available in our healthcare jurisdiction, to optimize the capture of AF recurrence. This approach may have underestimated the rate of recurrence, as compared to the gold standard of continuous implanted monitors. However, the generalizability of the results of this study is limited due to its retrospective single-centre design. The slow elimination rate of amiodarone may limit detection of AF recurrence on continuous ambulatory ECG monitors. Following chronic therapy cessation, amiodarone is observed to have a half-life of 13-142 days as tissue stores are depleted.^28^ Given a median time of 72 days between hospital discharge and continuous ambulatory ECG monitoring, residual antiarrhythmic effects of amiodarone may have influenced the rate of AF recurrence seen on ECG monitoring. Consequently, we may have underestimated the incidence of AF recurrence.

### Conclusions

A dedicated outpatient clinic for follow-up care of patients with POAF following cardiac surgery hospitalization documented AF recurrence in approximately 1 in 10 patients. For patients with POAF, use of a 14-day continuous ambulatory ECG monitor may be an effective strategy for identifying the subset of patients with AF recurrence who will benefit from evidence-based therapies for AF.
